# Impact of human sepsis on CCCTC-binding factor associated monocyte transcriptional response of Major Histocompatibility Complex II components

**DOI:** 10.1371/journal.pone.0204168

**Published:** 2018-09-13

**Authors:** Benedikt Hermann Siegler, Florian Uhle, Christoph Lichtenstern, Christoph Arens, Marek Bartkuhn, Markus Alexander Weigand, Sebastian Weiterer

**Affiliations:** 1 Department of Anesthesiology, Heidelberg University Hospital, Im Neuenheimer Feld 110, Heidelberg, Baden-Württemberg, Germany; 2 Institute for Genetics, Justus-Liebig-University Giessen, Heinrich-Buff-Ring 58–62, Giessen, Hessen, Germany; "INSERM", FRANCE

## Abstract

**Background:**

Antigen presentation on monocyte surface to T-cells by Major Histocompatibility Complex, Class II (MHC-II) molecules is fundamental for pathogen recognition and efficient host response. Accordingly, loss of Major Histocompatibility Complex, Class II, DR (HLA-DR) surface expression indicates impaired monocyte functionality in patients suffering from sepsis-induced immunosuppression. Besides the impact of Class II Major Histocompatibility Complex Transactivator (CIITA) on MHC-II gene expression, X box-like (XL) sequences have been proposed as further regulatory elements. These elements are bound by the DNA-binding protein CCCTC-Binding Factor (CTCF), a superordinate modulator of gene transcription. Here, we hypothesized a differential interaction of CTCF with the MHC-II locus contributing to an altered monocyte response in immunocompromised septic patients.

**Methods:**

We collected blood from six patients diagnosed with sepsis and six healthy controls. Flow cytometric analysis was used to identify sepsis-induced immune suppression, while inflammatory cytokine levels in blood were determined via ELISA. Isolation of CD14^++^ CD16^—^monocytes was followed by (i) RNA extraction for gene expression analysis and (ii) chromatin immunoprecipitation to assess the distribution of CTCF and chromatin modifications in selected MHC-II regions.

**Results:**

Compared to healthy controls, CD14^++^ CD16^—^monocytes from septic patients with immune suppression displayed an increased binding of CTCF within the MHC-II locus combined with decreased transcription of *CIITA* gene. In detail, enhanced CTCF enrichment was detected on the intergenic sequence XL9 separating two subregions coding for MHC-II genes. Depending on the relative localisation to XL9, gene expression of both regions was differentially affected in patients with sepsis.

**Conclusion:**

Our experiments demonstrate for the first time that differential CTCF binding at XL9 is accompanied by uncoupled MHC-II expression as well as transcriptional and epigenetic alterations of the MHC-II regulator CIITA in septic patients. Overall, our findings indicate a sepsis-induced enhancer blockade mediated by variation of CTCF at the intergenic sequence XL9 in altered monocytes during immunosuppression.

## Introduction

Antigen presentation on monocyte surface to CD4^+^-T-lymphocytes by Major Histocompatibility Complex, Class II (MHC-II) molecules is known as an essential mechanism for pathogen recognition and the subsequent initiation of an efficient host response [1]. The significance of this process becomes apparent during sepsis—one of the most life-threatening complications in intensive care medicine—where functional impairment of antigen-presenting cells contributes to the phenomenon of immune paralysis and exposes the patients to a high risk for opportunistic infections [2, 3]. Among these cells, classical monocytes (CD14^++^ CD16^-^) represent the largest subpopulation with high capacity of phagocytosis [4]. As a pathogen recognition receptor (PRR), the glycoprotein CD14 specifically recognizes pathogen associated molecular patterns (PAMPs) like LPS [5]. In addition, after *in vitro* LPS stimulation, CD14^++^ CD16^-^ monocytes impress by producing a broad range of different cytokines including CC-chemokine ligand 2 (CCL2) and interleukin (IL) 10 [4] and seem to be relevant in the initial sepsis-induced immune response [6]. Moreover, decreased monocyte surface expression of Major histocompatibility Complex, Class II, DR (HLA-DR) has been identified as a reliable surrogate of global immunosuppression serving as an independent predictor of mortality in this critically ill population [7]. Importantly, it has been shown that in patients with low HLA-DR values, only CD14^++^ CD16^-^ monocytes showed a greatly reduced HLA-DR expression while CD16^+^ cells continued to express HLA-DR on their surface. This indicates that MHC-II regulation is particularly affected in CD14^++^ CD16^-^ cells during immunosuppression [8].

Regulation of the cellular mechanisms required for efficient pathogen elimination including antigen presentation is exceedingly complex and in parts takes place at the level of gene transcription [9]. Genes encoding for chain components of MHC-II proteins are located in a dense cluster of human chromosome 6 and underlie transcriptional control by the master regulator Class II Major Histocompatibility Complex Transactivator (CIITA) [9, 10]. While CIITA coordinates the interaction between a complex network of DNA-binding factors and conserved *cis*-acting W-X-Y-boxes in the promoters of MHC-II genes, the existence of further regulatory components was proposed after identifying DNA regions with homology to X-box sequences inside the HLA-DR subregion of the MHC-II locus, designated as X box-like (XL) elements [11].

Among these, a highly acetylated sequence located in a ~53 kb spanning intergenic region separating the diametrically transcribed genes *Major Histocompatibility Complex*, *Class II*, *DR Beta 1* and *DQ Alpha 1* (*HLA-DRB1* and *HLA-DQA1*) has been discovered and termed XL9 [12]. Whereas XL9 is not directly bound by CIITA and neither seems to function as an enhancer or repressor, a potent enhancer-blocking activity could be revealed by experiments in different cell lines including Burkitt`s lymphoma-derived Raji and HS-Sultan cells [12]. Furthermore, a core sequence of XL9 displaying a peak of histone 3 (H3) acetylation was detected as a binding site for the highly conserved mammalian DNA-binding protein CCCTC-Binding Factor (CTCF), a superordinate regulator [13]. CTCF contains a central 11-zinc finger domain allowing the protein to bind approximately 20.000–40.000 sites in the human genome [14–16]. Aside from its enhancer blocking capability and other modulatory functions influencing gene transcription, CTCF is further known as a chromatin insulator with impact on the global organization of chromatin architecture and topology via loop formation [13].

In previous work, we performed genome-wide histone modification analysis of human CD14^++^ CD16^—^ monocytes demonstrating that human sepsis results in selective and precise changes of chromatin modifications in distinct promoter regions of immunologically relevant genes, including those inside the MHC-II locus [17]. Here, we hypothesized a transcriptional modulation of the MHC-II locus with differential interaction of the zinc finger protein CTCF. For the first time, our study reveals reduced expression of MHC-II- and *CIITA* genes associated with specific changes in binding of CTCF in the intergenic region in critically ill patients suffering from sepsis.

## Materials and methods

### Ethics and patient enrolment

All experiments were performed in accordance with the principles expressed in the Declaration of Helsinki and with the approval of the ethics committees of the medical faculty of the Justus-Liebig-University of Giessen (Klinikstrasse 32, D-35385 Giessen, Germany; approval number 155/12) and the medical faculty of the Heidelberg University (Alte Glockengießerei 11/1, D-69115 Heidelberg, Germany; approval number S-135/2016). All subjects had to be at least 18 years old. Exclusion criteria comprised participation in an interventional study, infectious viral diseases (HIV, hepatitis) as well as pre-existing renal failure, auto-immune diseases or immune-suppressive medication. Sepsis had to be diagnosed (according to reference [18]) within 24 hours prior to study inclusion.

### Quantitative HLA-DR detection on monocyte surface

Flow cytometry using a FACSCalibur flow cytometer (BD Bioscience, Heidelberg, Germany) was performed to determine HLA-DR expression on CD14^++^-monocytes. According to manufacturer`s instructions, ETDA-anti-coagulated whole blood was incubated with anti-HLA-DR antibody (Quantibrite anti-HLA-DR/Monocyte antibody, BD Bioscience, Heidelberg, Germany) followed by erythrocyte lysis (FACS Lysing solution, BD Bioscience, Heidelberg, Germany). Daily measured 4-point calibration curves (Quantibrite PE Beads, BD Bioscience, Heidelberg, Germany) allowed the conversion of determined sample values to molecules of HLA-DR on monocyte surface.

### Cell isolation of CD14^++^ CD16^—^monocytes

Monocyte separation was conducted as described previously [19]. Peripheral blood mononuclear cells were isolated from a 30 ml Lithium-heparin-anti-coagulated blood sample by Ficoll-based density gradient centrifugation. Magnetic cell sorting (autoMACS, Miltenyi Biotec, Bergisch Gladbach, Germany) using CD14 and CD16 MicroBeads (Miltenyi Biotec, Bergisch Gladbach, Germany) was performed to deplete CD16^++^ monocytes and to isolate CD14^++^ CD16^—^monocytes, followed by flow cytometry after incubation with FITC anti-human CD14 Antibody (BioLegend, San Diego, USA) to ensure purity of the isolated cells.

### Gene expression analysis

For gene expression analysis, RNA was extracted from CD14^++^ CD16^—^monocytes using a column-based method (RNeasy Plus Mini Kit, Qiagen, Hilden, Germany) according to manufacturer`s instructions. RNA concentration and quality were determined via spectrophotometry (Nanodrop, Thermo Fisher Scientific, Waltham, USA). Both 260/280 as well as 260/230 ratios of RNA samples used for reverse transcription were above 1.8. Reverse transcription was conducted using 1 μg RNA according to manufacturer`s instructions (Quantitect Reverse Transcription Kit, Qiagen, Hilden, Germany). Quantitative PCR was performed on a StepOnePlus PCR-cycler (Applied Biosystems, Foster City, USA) using commercial TaqMan assays (Applied Biosystems, Foster City, USA; S1 Table) and reagents (TaqMan Gene Expression Master Mix, Applied Biosystems, Foster City, USA) with all reactions done in triplicate. ΔCt analysis was conducted by subtraction of the values of the gene of interest from the mean of the two endogenous control genes *Actin Beta* (*ACTB*) and *Hypoxanthine Phosphoribosyltransferase 1* (*HPRT1*). The relative gene expression was then calculated by 2^ΔCt^.

### Chromatin immunoprecipitation and quantitative PCR analysis

Chromatin immunoprecipitation (ChIP) was conducted as previously published [19]. Using formaldehyde (final concentration 1%), isolated CD14^++^ CD16^—^monocytes were cross-linked for 10 minutes at 18°C. Glycine (final concentration 0.125 M) stopped the fixation. Cell lysis (10^6^ cells per 200 μl lysis buffer) was followed by sonication with Branson sonifier (Branson Ultrasonics, Danbury, USA; fragment size 150–600 base pairs) under constant cooling. Immunoprecipitation was performed using agarose A/G-beads (Merck, Darmstadt, Germany). To mix chromatin and antibodies (obtained from Diagenode, Liège, Belgium, for details see S1 Table) for acetylated lysine 9, acetylated lysine 27 or trimethylated lysine 27 of histone 3 (H3K9ac, H3K27ac and H3K27me3) as well as CTCF, samples were placed in a rotator for 3 hours at 4°C. Before reversal of cross-links by incubation for several hours at 65°C (with additional supplementation of RNase A followed by 10% SDS/Proteinase K mix), chromatin bead complex was cleared using different concentrations of salt wash buffers. DNA purification was conducted via Illustra GFX PCR DNA and Gel Band Purification kit (GE Healthcare Life Science, Freiburg, Germany) followed by elution in pure H_2_O. For quality control, the amount of ChIP-DNA was measured using a Qubit assay according to manufacturer`s instructions (Qubit Fluorometric Quantitation, Thermo Fisher Scientific, Waltham, USA). Specific primer sets for *CIITA*, the intergenic element XL9 and MHC-II genes as well as *MB* (*Myoglobin*) and H19-ICR (H19/IGF2 Imprinting Control Region) were used for subsequent quantitative PCR analysis using a StepOnePlus PCR-cycler (Applied Biosystems, Foster City, USA). Primer sequences are listed in S1 Table. The XL9 amplicon spans an acetylated fragment of XL9 previously identified as a specific CTCF binding site [12].

### Enzyme-linked immunosorbent assay

Plasma was collected after centrifugation of whole blood and used for IL-6 and tumor necrosis factor α (TNF-α) measurement by enzyme-linked immunosorbent assay (ELISA) according to manufacturer`s instructions (R&D Technologies, North Kingstown, USA).

### Data visualization and statistical analysis

Visualization of the data and statistical analysis was performed using GraphPad Prism (Version 6.0f, GraphPad Software, La Jolla, USA). Results were visualized as bars and expressed as mean and standard error of the mean (SEM). Mann Whitney U-test was used to compare experimental data of patients and healthy controls. P-values less than 0.05 were considered significant and indicated with ‘*’ (p<0.05, >0.01) or ‘**’ (p<0.01).

## Results

### Patients' characteristics, cytokine levels and flow cytometry

**Fig 1:**
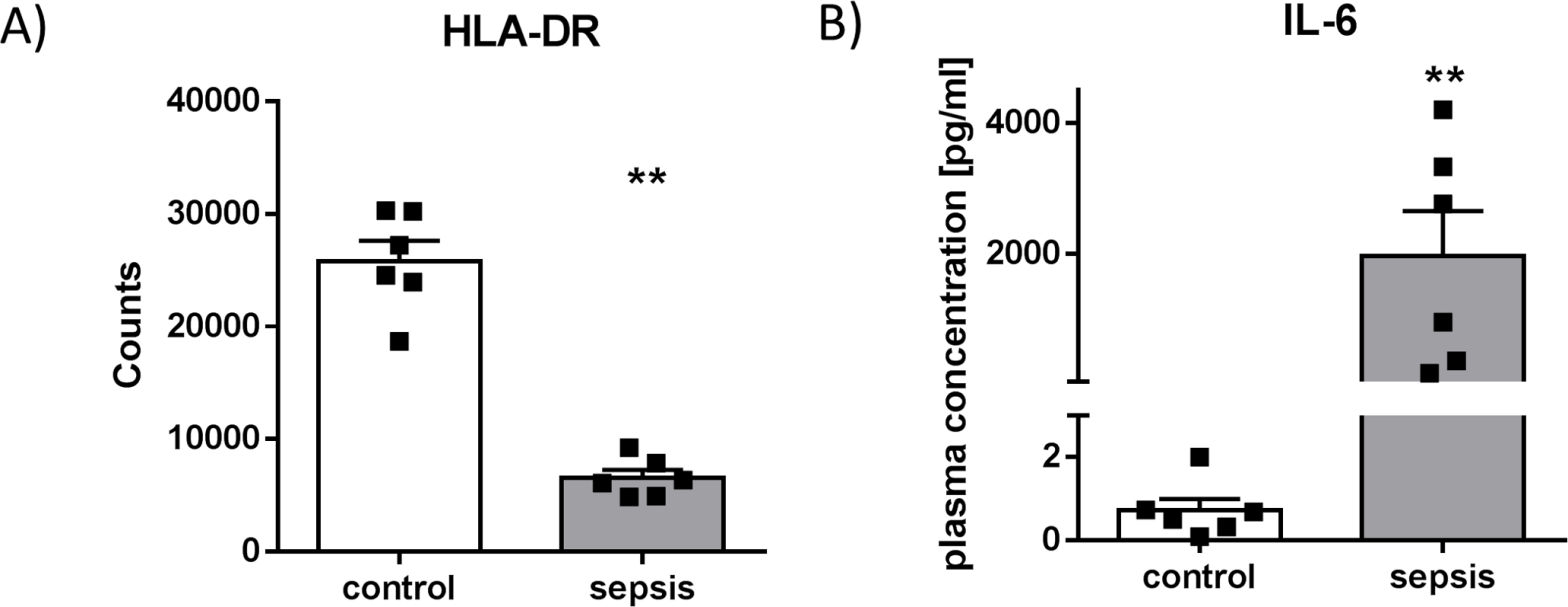
HLA-DR expression on monocytes and plasma levels of IL-6. (A) HLA-DR expression in sepsis. Surface expression of HLA-DR on CD14^++^-monocytes analyzed in whole blood samples from patients with sepsis and healthy controls using flow cytometry. (**p<0.01, Mann Whitney U-test, control n = 6, patients with sepsis n = 6, Mean + SEM). (B) IL-6 levels in sepsis. (**p<0.01, Mann Whitney U-test, control n = 6, patients with sepsis n = 6, Mean + SEM).

Six patients with clinical diagnosis of sepsis and six healthy donors were included. Clinical characteristics of the study collective are summarized in S2 Table. Blood samples were taken to identify a low expression of HLA-DR on monocytes via flow cytometry, indicating a suppressive immune phenotype. Healthy volunteers showed an average of 25823 HLA-DR molecules (18696–30292 molecules) compared to an average of 6580 molecules per monocyte from patients with immunosuppressive sepsis (4876–9259 molecules, **p<0.01) (Fig 1A). In addition, ELISA measurements revealed strongly increased plasma levels of IL-6 in the investigated group of septic patients (**p<0.01) (Fig 1B), while TNF-α was not detectable in both collectives (S1 Fig).

### Differential expression and distribution of histone modifications at the promoter region of CIITA gene

**Fig 2:**
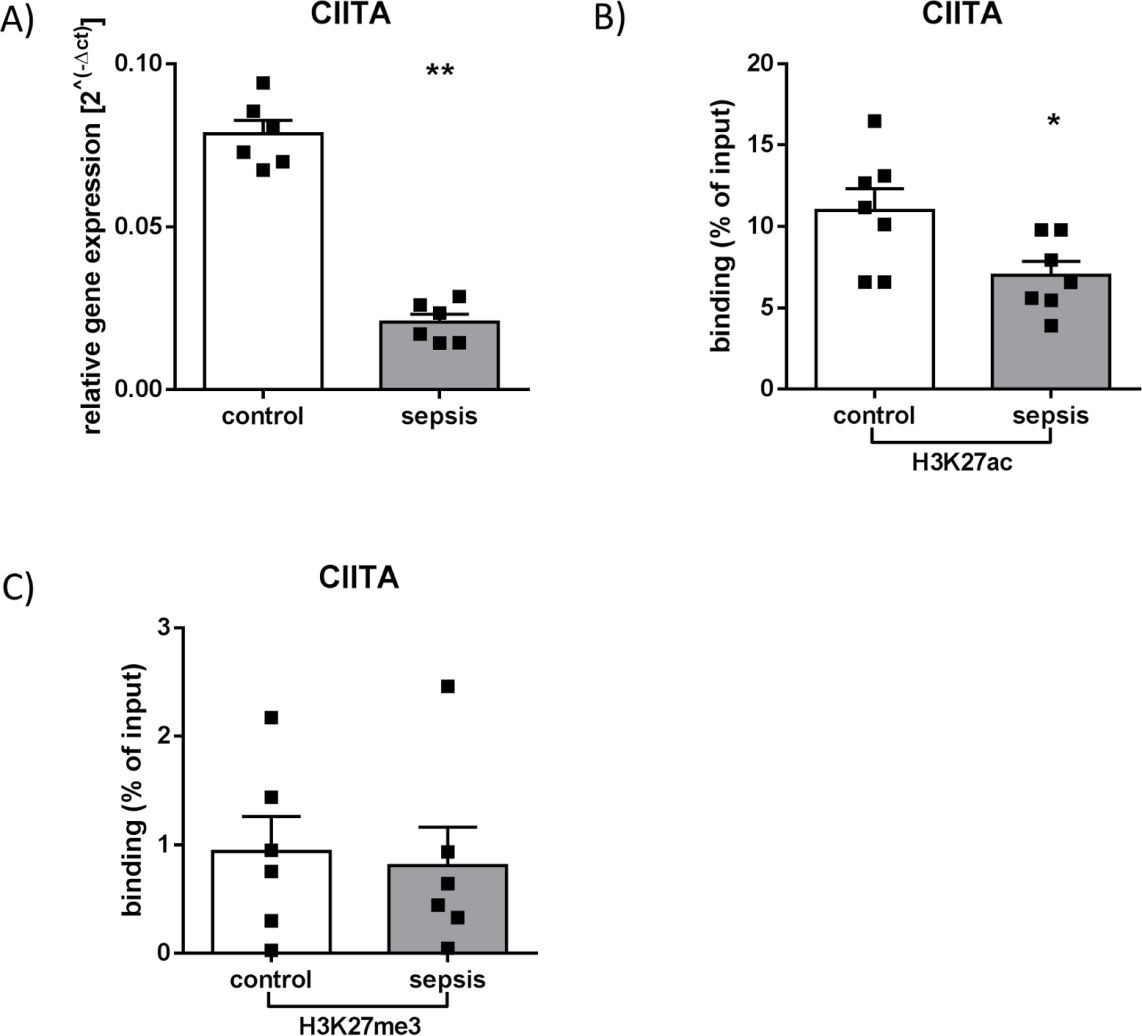
Expression and chromatin modifications in the promoter region of transcriptional co-activator *CIITA* gene. (A) Expression of *CIITA* gene. RNA from isolated human CD14^++^ CD16^—^monocytes was used for reverse transcription and subsequent qPCR using TaqMan Assay against CIITA **p<0.01, Mann Whitney U-test, control n = 6, patients with sepsis n = 6, Mean + SEM). (B) H3K27ac and (C) H3K27me3 binding on promoter regions of *CIITA* gene. Chromatin from isolated human CD14^++^ CD16^—^monocytes was immunoprecipitated with anti-H3K27me3 and anti-H3K27ac antibodies for subsequent qPCR using primer pairs specific on target region *CIITA*. (*p<0.05, Mann Whitney U-test, control n = 6, patients with sepsis n = 6, Mean + SEM).

Gene expression analysis from RNA of isolated human CD14^++^ CD16^—^monocytes showed a decreased expression of the gene coding for the master regulator of the MHC-II locus on chromosome 6, *CIITA*, in septic patients with a HLA-DR suppressive phenotype (**p<0.01) (Fig 2A). This was accompanied by a significantly lower enrichment of H3K27ac in the promoter region of *CIITA* (*p<0.05), a modification predominantly associated with a higher transcriptional activity (Fig 2B). ChIP experiments also revealed an overall low enrichment of H3K27me3. In addition, no differences of this per se inactive mark could be detected in the *CIITA* promoter region of patients with sepsis compared to healthy controls (p = 0.57) (Fig 2C).

### Sepsis-induced changes of CTCF binding and acetylation enrichments on XL9

**Fig 3:**
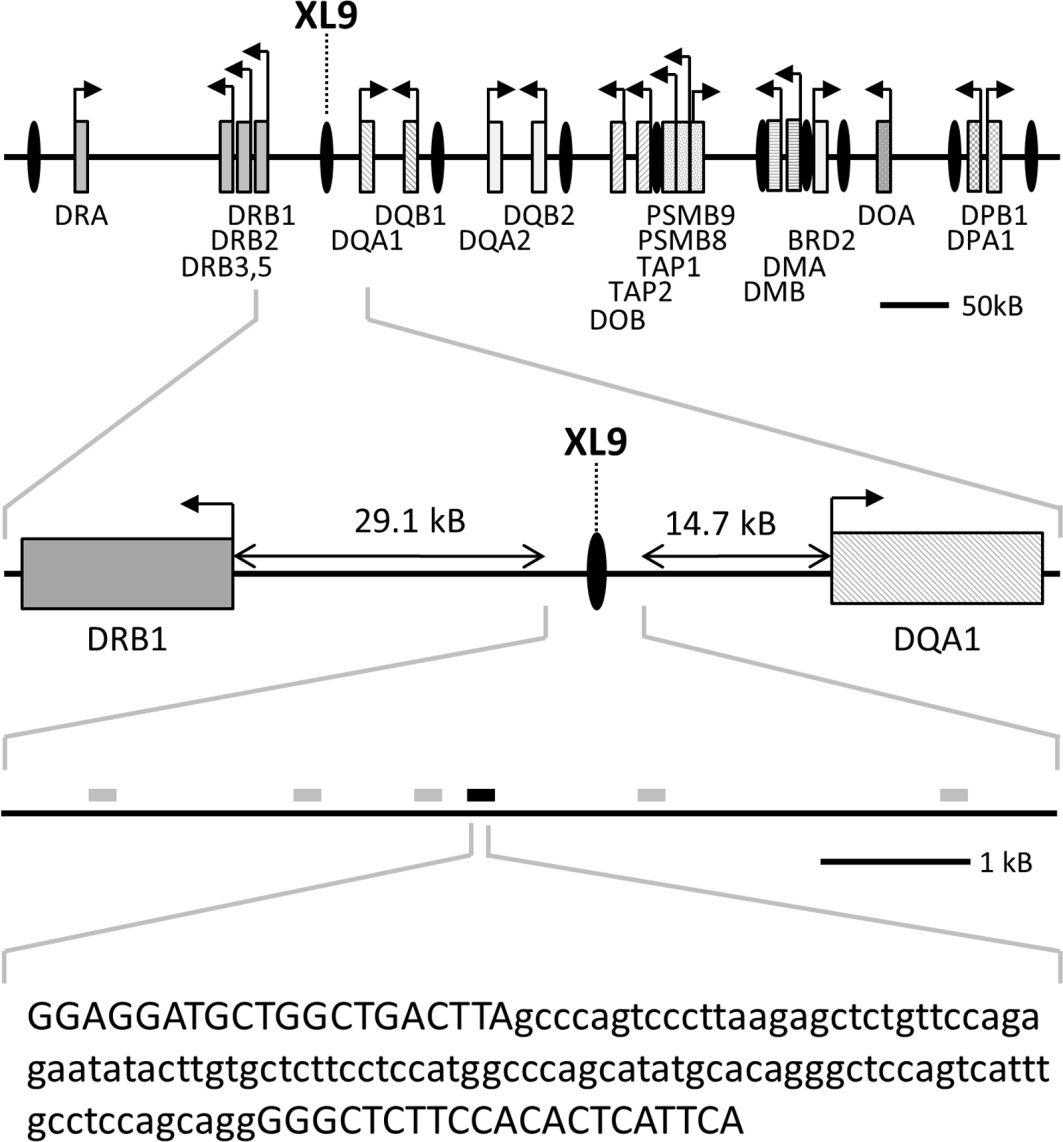
CTCF-binding sites and localization of XL9 inside the MHC-II locus. Schematic diagram showing the relative positions of MHC-II genes (grey shaded boxes) and CTCF binding sites (black ovals) on human chromosome 6p21.3 as identified by Majumder and Boss [20] with the XL9 region flanked by *HLA-DRB1* and *HLA-DQA1* (upper 2 panels). Grey and black bars show identified areas of the XL9 region associated with highly acetylated histones—the black bar shows a confirmed specific CTCF binding sequence [12]. The latter is encompassed by the primer set amplicon used in our study (lower 2 panels).

**Fig 4:**
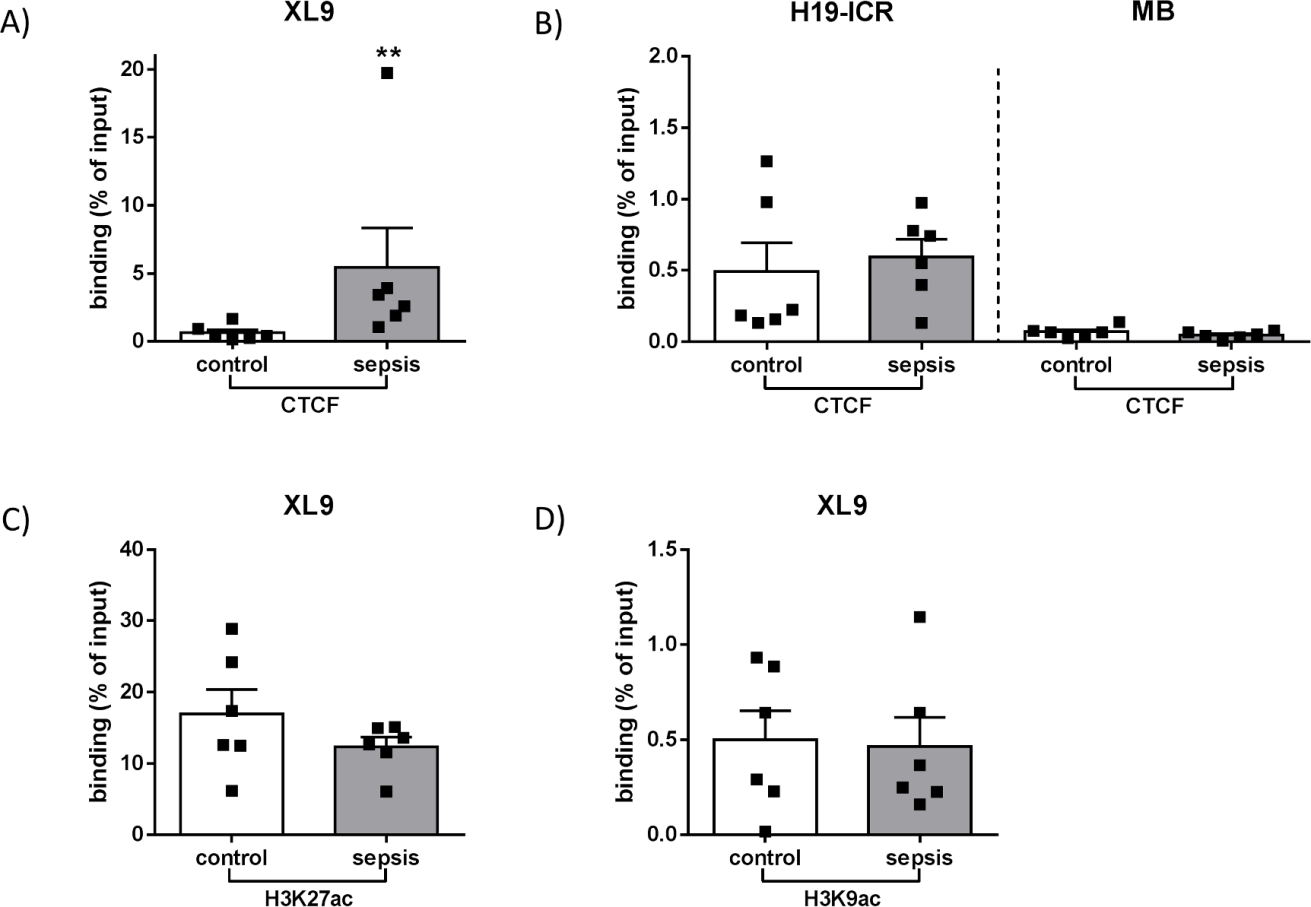
Binding of CTCF and acetylated histones on the intergenic element XL9. Chromatin from isolated human CD14^++^ CD16^—^monocytes was immunoprecipitated with anti-CTCF antibody for subsequent qPCR using primer pairs on specific target regions. (A) Enrichment of CTCF at XL9 (**p<0.01, Mann Whitney U-test, control n = 6, patients with sepsis n = 6, Mean + SEM). (B) CTCF binding at control sites H19-ICR and *MB*. Two Selected genome regions served as positive (H19-ICR, chromosome 11) and negative (*MB*, chromosome 22) controls for CTCF binding (p>0.05, Mann Whitney U-test, control n = 6, patients with sepsis n = 6, Mean + SEM). (C) and (D) Chromatin acetylation in the XL9 region. Chromatin from isolated human CD14^++^ CD16^—^monocytes was immunoprecipitated with anti-H3K27ac and anti-H3K9ac antibody for subsequent qPCR using primer pairs specific on target region XL9 (p>0.05, Mann Whitney U-test, control n = 6, patients with sepsis n = 6, Mean + SEM).

Further investigations of the MHC- II locus via ChIP-experiments and subsequent qPCR showed an elevated binding of CTCF at the boundary between HLA-DR and HLA-DQ subregion of CD14^++^ CD16^—^monocytes derived from patients with FACS-confirmed immunosuppression (**p<0.01). In particular, enhanced CTCF enrichment was detected on a distinct region known as XL9, which is located between the two diametrically transcribed MHC-II genes *HLA-DRB1* and *HLA-DQA1* (Figs 3 and 4A). Since there was an outlier with a high numerical value (19.7%), further analysis was performed to confirm that it did not determine the statistical difference of CTCF binding at XL9 between both groups (p = 0.0043 (**) vs. p = 0.0087 (**) without outlier). A Spearman correlation test used to determine whether the increased CTCF binding at XL9 was linked to the observed reduction in CIITA expression revealed no correlation between CIITA expression and CTCF binding in the intergenic region during immune suppressive sepsis (R square = 0.2633; p = 0.08).

Additional PCR experiments were performed using optimized primers for specific regions as negative and positive controls for CTCF binding (negative control: *MB*; positive control: H19-ICR), both located outside the observed MHC-II locus. There was no relevant CTCF-binding inside the *MB* gene and neither the negative nor the positive control region differed in their CTCF enrichments comparing healthy controls and sepsis patients, respectively (p = 0.31 and p = 0.79) (Fig 4B). To determine whether changes in the distribution of histone modifications could explain the differential binding of CTCF at the XL9 sequence, ChIP-experiments were performed using antibodies against H3K9ac and H3K27ac, marks associated with an open chromatin structure. Sepsis was not associated with altered acetylation of lysine 9 (p = 0.90) and lysine 27 (p = 0.47) of histone 3 with a high overall enrichment of H3K27ac in both groups (Fig 4C and 4D).

### Differential impact of sepsis on HLA-DR and HLA-DQ subregions surrounding XL9

**Fig 5:**
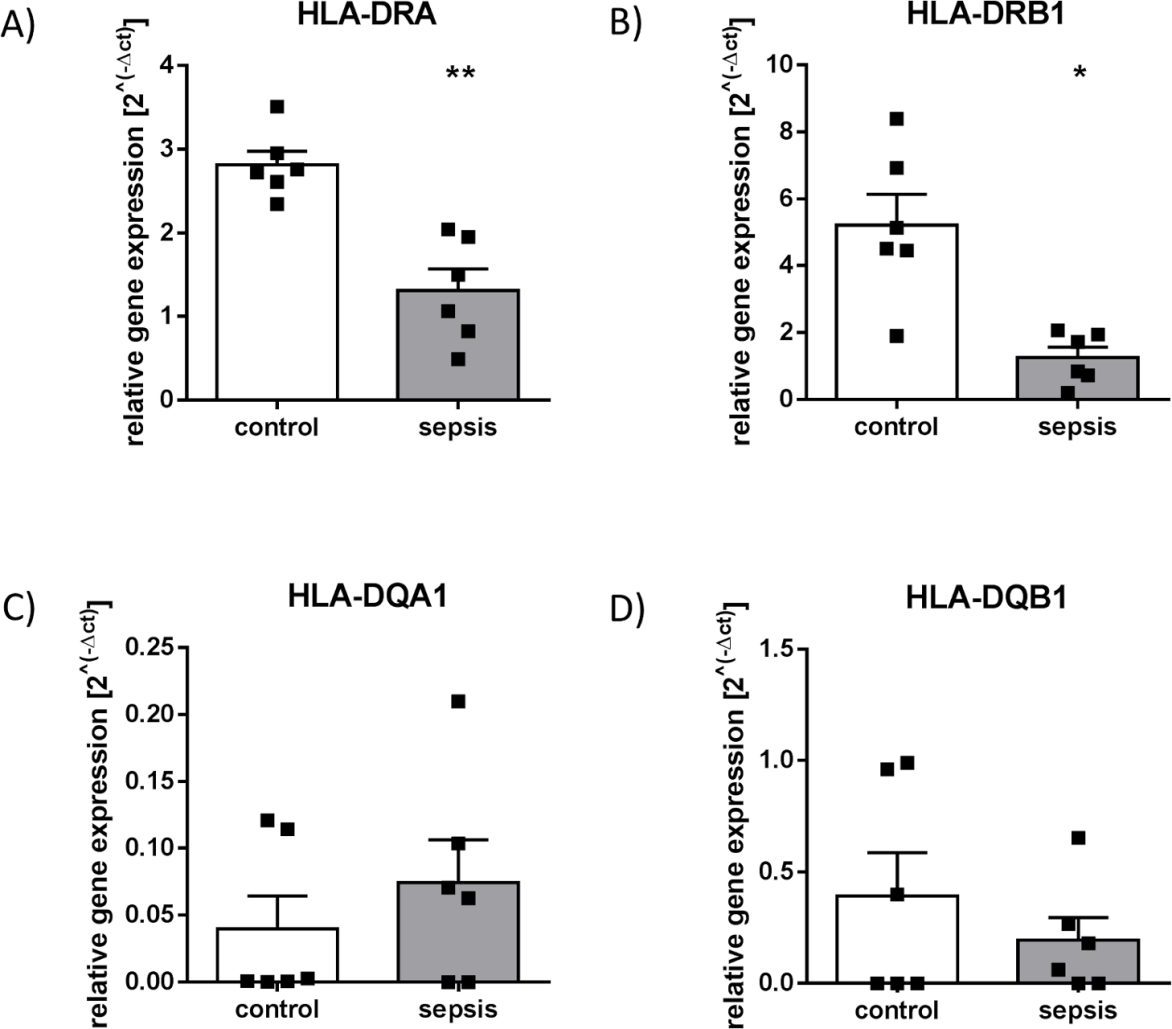
Expression of MHC-II genes encoding for different HLA isotypes. RNA from isolated human CD14^++^ CD16^—^monocytes was used for reverse transcription and subsequent qPCR experiments using TaqMan Assay against HLA-DRA (A), HLA-DRB1 (B), HLA-DQA1 (C) and HLA-DQB1 (D) (*p<0.05, **p<0.01, Mann Whitney U-test, control n = 6, patients with sepsis n = 6, Mean + SEM).

**Fig 6:**
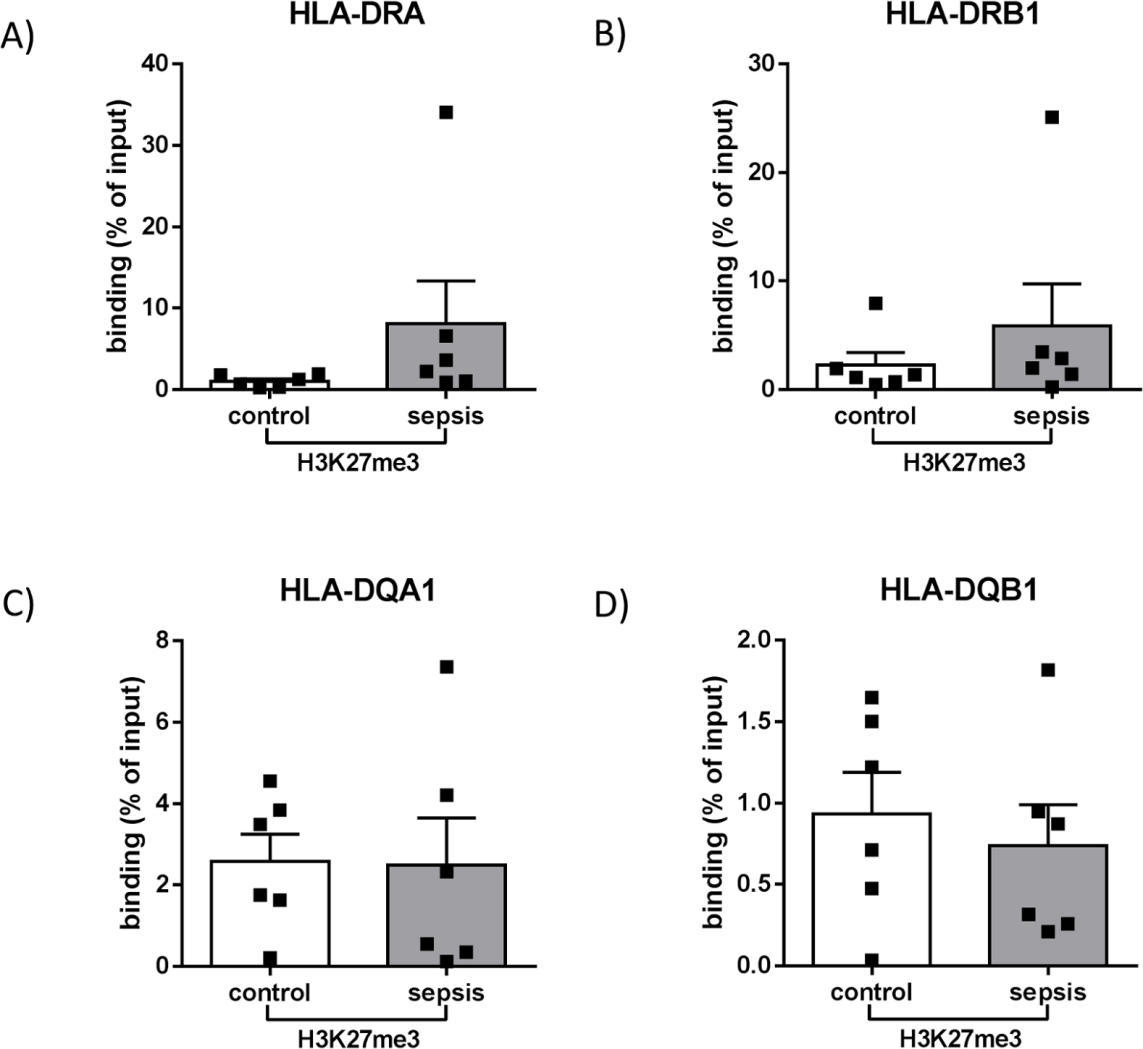
H3K27me3 binding in the promoter regions of human MHC-II genes. Chromatin from isolated human CD14^++^ CD16^—^monocytes was immunoprecipitated with anti-H3K27me3 antibody for subsequent qPCR using primer pairs specific on target regions *HLA-DRA* (A), *HLA-DRB1* (B), *HLA-DQA1* (C) and *HLA-DQB1* (D) (p>0.05, Mann Whitney U-test, control n = 6, patients with sepsis n = 6, Mean + SEM).

Expression of genes placed adjacent to the X-box like element was differentially affected in patients with sepsis compared to healthy control patients depending on their localization relative to XL9. In detail, genes encoding for subunits of HLA-DR (*HLA-DRA* and *HLA-DRB1*) showed a lower expression in patients with HLA-DR suppressive sepsis (**p<0.01 and *p<0.05) (Fig 5A and 5B). ChIP-experiments with antibodies against the repressive histone modification H3K27me3 revealed that lower gene expression of *HLA-DRA* and *HLA-DRB1* was not associated with H3K27me3 enrichment over both HLA-DR gene regions (p = 0.06 and p = 0.31) (Fig 6A and 6B). In contrast to the investigated HLA-DR genes, no significant sepsis-induced changes were detected in transcription of *HLA-DQA1* and *HLA-DQB1* (p = 0.81 and p>0.99) (Fig 5C and 5D). Moreover, distribution of H3K27me3 within the promoter regions of HLA-DQ genes did not differ compared to healthy controls (p = 0.79 and p = 0.68) (Fig 6C and 6D).

## Discussion

CTCF has been recognised as an important genome-wide regulator of gene expression in a variety of cell types including phagocytes; however, its specific impact on antigen-presentation in sepsis-induced immune suppression has not been investigated so far. In this study, we analysed CD14^++^ CD16^—^monocytes derived from an immune-compromised septic collective and could demonstrate for the first time that CTCF-binding at the highly acetylated intergenic sequence XL9 is elevated in these patients compared to healthy controls. Moreover, expression of HLA components was differentially affected in the investigated group of septic patients dependent on the localization of the corresponding genes relative to XL9. In contrast to non-effected HLA-DQ genes, genes of the HLA-DR subregion exhibited lower expression levels in sepsis. Since XL9 was identified as a CTCF-binding site and may thus function as potential enhancer-blocking sequence [12], our findings support the hypothesis that this region represents a relevant regulatory element of the MHC-II locus modulated by CTCF in immune-compromised patients suffering from sepsis.

Sepsis-induced paralysis of the immune system—mainly based on dysfunctional and apoptotic lymphatic and myeloid cells—renders the patients susceptible to opportunistic infections and significantly contributes to reduced survival rates; therefore several biomarkers for immune-monitoring have been investigated [2, 3, 21–23]. Among these, diminished HLA-DR surface expression on monocytes represents a reliable indicator of global immunosuppression [24, 25]. Beside high plasma levels of IL-6, which serves as a fast responding sepsis marker correlating with disease severity and exhibits prognostic value concerning 28-day mortality [26–28], our patient collective was characterized by low HLA-DR values indicating a loss of monocyte functionality regarding antigen presentation to T-cells.

The process of pathogen recognition is tightly controlled and involves conserved *cis*-acting W-X-Y elements located near the transcriptional start sites of MHC-II genes on chromosome 6p21.3 [9, 10, 29, 30]. While X- and Y-boxes are bound by a heteromultimer including Regulatory Factor X (RFX), the CAMP Responsive Element Binding Protein (CREB) and the Nuclear Transcription Factor Y (NFY), expression of MCH-II genes further requires CIITA as a superordinate coordinator of transcriptional and chromatin remodelling machinery [9, 10]. The essential role of the master regulator of MHC-II gene expression has been emphasised by studies showing that altered CIITA expression contributes to neoplastic diseases and immune disorders [31, 32].

Furthermore and in accordance with our results, it is known that loss of monocyte HLA-DR surface expression during sepsis is at least in part regulated on the transcriptional level. This was argued from experiments showing both a decrease in HLA-DR mRNA levels and a reduced transcription of *CIITA* [33]. Nonetheless, a possible superordinate regulation including epigenetic mechanisms of MHC-II in monocytes of septic patients had not been investigated. However, in a previous study, we could demonstrate specific changes in histone modifications and found sepsis-induced alterations in both MHC-II locus and *CIITA* gene of isolated CD14^++^ CD16^—^monocytes [17]. Here, we provide additional insights into sepsis-induced chromatin variations, which are associated with reduced expression of MHC-II- and *CIITA* genes in these antigen-presenting cells, a finding that underscores the involvement of a much more comprehensive regulatory system controlling transcription of MHC-II components during sepsis. The complexity of this system is further raised by the multifaceted roles of CIITA: Complementary to its transactivator function as a member of an enhanceosome complex containing RFX, CREB and NFY, it has further been shown to acetylate histone 3 and 4 as a putative mechanism to keep MHC-II chromatin accessible for transcription [34].

Aside from CIITA, the zinc finger protein CTCF has been discovered as superordinate regulator of gene expression with several binding sides spread across the MHC-II locus ([20]). The insulator protein initiates long-range chromatin loops thereby blocking the activity of adjacent enhancers [35]. Recent studies indicate CTCF to be essential for maximal transcription of MHC-II genes in murine macrophages and B cells [36, 37]. Interestingly and in contrast to the mouse, CTCF binding sites in the human MHC-II locus are located in boundary regions, where they separate genes coding for HLA isotype-specific α- and ß-chain components [20, 36]. Among these binding sites, cell culture experiments using lymphoma B cells identified an X box-like element named XL9 between the two diametrically transcribed genes *HLA-DRB1* and *HLA-DQA1* [12].

Within this ~53 kB spanning intergenic region, which seems to play an important role in the pathogenesis of autoimmune diseases [38], the XL9 sequence is characterised by high acetylation levels—as confirmed by our results—and does not show an association with CIITA or the DNA-binding factor RFX [12]. While in general, transcription of HLA-DR- and HLA-DQ-isotypes is modulated in a co-regulatory manner, different studies discuss XL9 itself as a potential enhancer blocking element, as uncoupled MHC-II gene activities up and downstream from XL9 have been detected in different tumour cell lines [39, 40] as well as in renal microvascular endothelium [41]. Accordingly, our findings demonstrate for the first time a sepsis-induced decoupled co-regulation of MHC-II gene transcription, which is linked to differential CTCF-XL9-interaction in immunocompromised and critically ill patients. Using primary CD14^++^ CD16^—^monocytes, ChIP-experiments revealed an elevated binding of CTCF on XL9, which was accompanied by decreased expression of genes coding for HLA-DR components, whereas transcription of HLA-DQ genes remained unaffected.

Interestingly, a previous study from Majumder et al. found that CTCF knockdown in MHC-II-expressing Raji cells, a B-cell line, resulted in a decrease of both *HLA-DRB1* and *HLA-DQA1* gene expression. In their work, a potential model of interaction between XL9, CIITA, the nuclear matrix, the RFX-CREB-NFY complex and the promoter regions of *HLA-DRB1* and *HLA-DQA1* mediated by CTCF has been proposed leading to active transcription of these genes [42]. The assumption of CTCF being critical for the expression of MHC-II genes is further strengthened by our study showing a basal occurrence of CTCF at XL9 in CD14++ CD16—monocytes of healthy control patients, that was accompanied by both HLA-DRB1 as well as HLA-DQA1 expression. However, during immune suppressive sepsis, expression levels of HLA-DR genes were significantly lower despite an even higher CTCF-XL9 binding compared to healthy controls. Hence, we speculate a significant change of the proposed interaction and a potential predominance of the insulator function of CTCF during sepsis. This idea of a disturbed CIITA—XL9—CTCF interaction in the critically ill patient is further supported by the observation that CIITA mRNA-expression was significantly reduced during immune suppressive sepsis (associated with decreased acetylation of lysine 27 of histone 3, which has previously been shown to play a role in regulating the *CIITA* gene [43, 44]). Remarkably, loss of CIITA mRNA-expression in the septic patients did not affect HLA-DQ gene expression. While CIITA is widely accepted as an important regulator mediating the assembly of DNA-binding factors at MHC-II promoters required for transcription [45, 46], our findings encourage the concept of a more complex regulatory network including CTCF determining MHC-II gene expression.

CTCF binding to DNA underlies a broad range of regulatory mechanisms [13]. However, it remains unclear what may cause the differential CTCF binding at XL9 during sepsis detected in our study. A recent analysis of ChIP-seq data obtained from resting human CD4^+^ T-lymphocytes as well as an epithelial cell line revealed a cell-type-specific chromatin architecture across the CTCF-binding sites, indicating a role of the positioning of surrounding nucleosomes on CTCF occupancy [47]. In general, insulator elements bound by CTCF are characterized by high levels of histone acetylation [48], which holds true for XL9 [12]. Since histone acetylation is commonly associated with open chromatin structure [49], we compared the enrichment of H3K9ac and H3K27ac at the investigated region of XL9 between the septic patient collective and the control group–however, the status of immune suppressive sepsis did not seem to have relevant influence on the levels of histone acetylation. This indicates that an increased binding of CTCF at XL9 during immune suppressive sepsis does not require an increase of acetylation at histone 3. In our view, this points towards other mechanisms that might control CTCF occupancy at XL9 during immune suppressive sepsis or inhibit further CTCF binding during a healthy state.

In summary, we found that differential CTCF binding at XL9 is accompanied by uncoupled MHC-II expression as well as transcriptional and epigenetic alterations of the MHC-II regulator CIITA in septic patients with proven HLA-DR reduction. Of note, all patients were included in our study within 24 hours after clinical diagnosis of sepsis. At that time, compensatory immune mechanisms might already co-exist, resulting in an overall refractory cellular phenotype [50]. In monocytes, this phenomenon becomes apparent during endotoxin tolerance experiments based on stimulation with TLR agonists [51]. However, whereas cell culture models can help to gain mechanistic insights, they roughly simulate the actual, complex in vivo situation during human immune compromised sepsis. Notably, the observed changes occurred despite both (i) different foci and (ii) varying causative pathogens of sepsis in our study collective, pointing towards a common, robust and specific MHC-II regulatory phenotype in immune-compromised CD14^++^ monocytes. Our results are in line with an earlier study uncovering decreased expression of MHC-II- and *CIITA* genes in peripheral whole blood samples from septic patients [33, 52] as well as own published work [17] and provide further insights on the impact of sepsis on monocyte MHC-II regulation.

As a limitation of our study, we did not perform age and gender matching of septic patients and healthy controls. Gender effects on the immune response during sepsis have been reported in humans as well as in animal models [53] including differences in inflammatory cytokine levels [54, 55] or immune cell counts [56]. With regard to the CD14++ CD16- subpopulation and the monocytic HLA-DR surface expression investigated in our study, a recent analysis revealed no impact of sex on the number of classical monocytes or the HLA-DR surface expression in the healthy population [57]. Moreover, HLA-DR expression seems not to be influenced by age, despite a slight increase in the number of classical monocytes in the elderly [57]. Since in our study the mean age did not differ and the male to female ratio was identical in healthy controls and patients with sepsis, we assume that not having matched for age and sex might only have minor impact on the results of our study.

## Conclusions

Overall, we discovered differential binding of the transcriptional insulator CTCF in the human MHC-II locus combined with selective changes in HLA-gene expression as well as transcriptional and epigenetic alterations of the MHC-II regulator CIITA gene in antigen-presenting cells of septic patients. Our findings indicate a sepsis-induced enhancer blockade mediated by variation of CTCF at the intergenic sequence XL9 as a potential mechanism contributing to an immunosuppressive phenotype in this critically ill patient population.

## Supporting information

S1 Table TaqMan assays and primers used for quantitative PCR analysis. (XLSX)

S2 Table Clinical characteristics of the study collective. (XLSX)

S3 Table Raw data of Figs 1–6 and S1 Fig. (XLSX)

S1 Fig TNF-α levels in patients with sepsis and healthy controls. (TIF)
