# Supplementary figures and images for: Impact of human sepsis on CCCTC-binding factor associated monocyte transcriptional response of Major Histocompatibility Complex II components
Source: PLoS One. 2018 Sep 13;13(9):e0204168. doi: 10.1371/journal.pone.0204168 (PMC6136812; doi:10.1371/journal.pone.0204168)

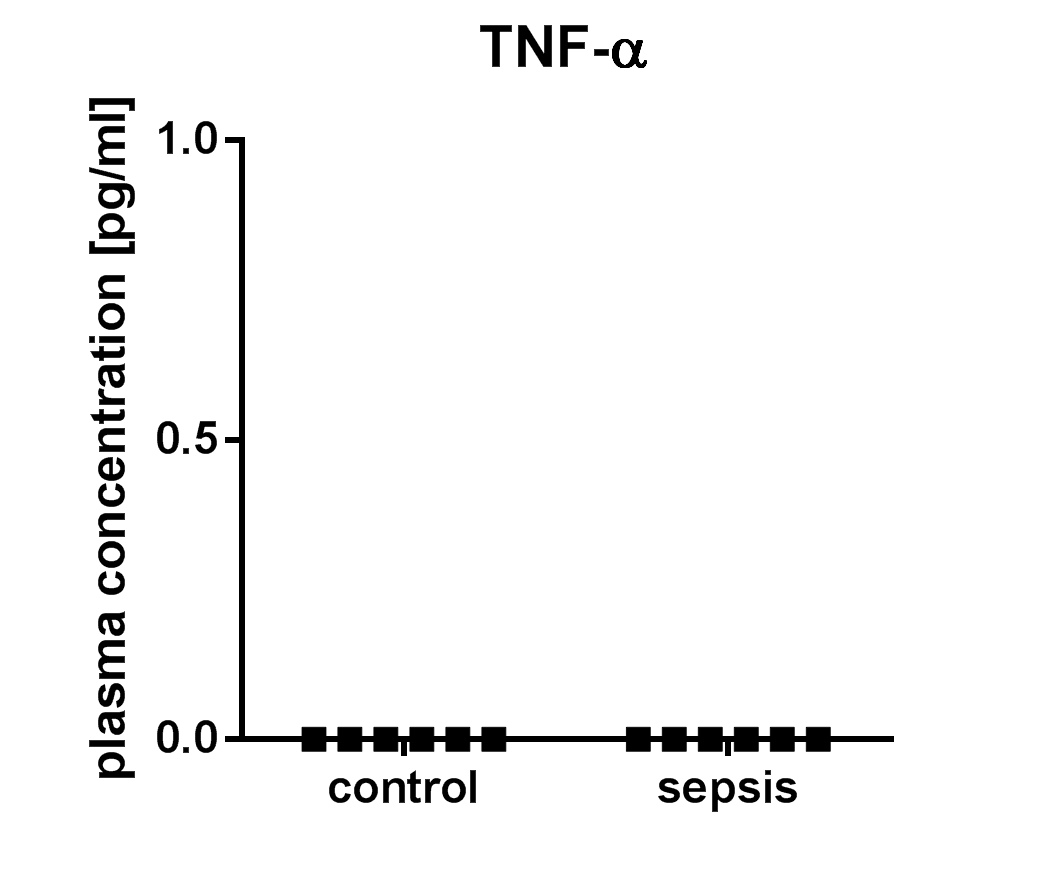

Supplement: S1 Fig — (TIF) [file pone.0204168.s004.tif]
